# Construction of a Chemical Kinetic Model of Five-Component Gasoline Surrogates under Lean Conditions

**DOI:** 10.3390/molecules27031080

**Published:** 2022-02-06

**Authors:** Chao Yang, Zhaolei Zheng

**Affiliations:** Key Laboratory of Low-Grade Energy Utilization Technologies and Systems, Ministry of Education, Chongqing University, Chongqing 400044, China; mryangchao@cqu.edu.cn

**Keywords:** cyclohexane, simplified chemical kinetic model, ignition delay time, laminar flame speed

## Abstract

The requirements for improving the efficiency of internal combustion engines and reducing emissions have promoted the development of new combustion technologies under extreme operating conditions (e.g., lean combustion), and the ignition and combustion characteristics of fuels are increasingly becoming important. A chemical kinetic reduced mechanism consisting of 115 species and 414 elementary reactions is developed for the prediction of ignition and combustion behaviors of gasoline surrogate fuels composed of five components, namely, isooctane, n-heptane, toluene, diisobutylene, and cyclohexane (CHX). The CHX sub-mechanism is obtained by simplifying the JetSurF2.0 mechanism using direct relationship graph error propagating, rate of production analysis, and temperature sensitivity analysis and CHX is mainly consumed through ring-opening reactions, continuous dehydrogenation, and oxygenation reactions. In addition, kinetic parameter corrections were made for key reactions R14 and R391 based on the accuracy of the ignition delay time and laminar flame velocity predictions. Under a wide range of conditions, the mechanism’s ignition delay time, laminar flame speed, and the experimental and calculated results of multi-component gasoline surrogate fuel and real gasoline are compared. The proposed mechanism can accurately reproduce the combustion and oxidation of each component of the gasoline-surrogate fuel mixture and real gasoline.

## 1. Introduction

Strict emission regulations place higher requirements on internal combustion engine technology (high efficiency and low emissions). Although the popularity of new energy vehicles with batteries as the main power continues to increase, research on hybrid power and the design of range extenders is inseparable from research on internal combustion engines. Clean fuels (e.g., alcohol and hydrogen) cannot completely replace traditional gasoline and diesel in the short-term. Selective catalyst reduction [1,2] to reduce NOx emissions and chemical kinetics to control in-cylinder combustion and heat transfer [3] have achieved certain results in diesel engines. In addition, new combustion technology research has also become a feasible method to improve engine efficiency and reduce emissions, such as homogeneous charge compression ignition [4], premixed charge compression ignition [5], and reactive control compression ignition (RCCI) [6]. However, these new gasoline engine technologies are increasingly becoming sensitive to the physical properties (e.g., density and viscosity) and chemical properties (e.g., flame propagation) of gasoline fuels [7]. Adding more real components of gasoline is necessary to ensure that gasoline-surrogate fuels match these physical and chemical properties.

The composition of gasoline-alternative fuels varies from single-component to multi-component. Single-component and binary alternatives can be used for simple applications [8,9]. Many alternative fuels with different physical and chemical properties have been considered. These alternatives include toluene blended fuel (TRF) composed of isooctane, n-heptane, and toluene [10,11,12], four-component fuel alternatives [13,14,15], and alcohol-containing alternatives [16,17], which have been verified in extensive experiments and model studies. Recently, naphthenic hydrocarbons have begun to be emphasized. Excluding olefins and naphthenic hydrocarbons may lead to inaccuracy in the prediction of gasoline-alternative fuels [18,19]. Andrae [18] found that adding CHX to a quaternary mixture including primary reference fuel (PRF), toluene, and diisobutylene (DIB) and partly replacing the PRF content increases the overall reactivity and improves model predictions at rapid compression machine (RCM) conditions. According to the study of Chu et al. [20], the addition of cycloalkane molecules to the laminar diffusion flame of n-heptane and isooctane promotes the formation of soot particles in the low-temperature region.

Cyclohexane (CHX), which is often used as a representative of cycloalkanes to study gasoline-surrogate fuels [18,21,22], has the simplest ring structure among cycloalkanes. Early studies on CHX were conducted in different reactors for CHX oxidation experiments [23,24]. Voisin et al. [23] conducted CHX oxidation studies in a jet stirrer reactor (JSR), and a chemical kinetic mechanism was proposed to verify its data. Subsequently, El-Bakali et al. [24] modified the mechanism of Voisin and expanded its validation range. Buda et al. [25] developed a detailed low-temperature oxidation chemical kinetic mechanism of CHX based on the EXGAS program, and this mechanism was able to satisfactorily reproduce experimental results obtained in an RCM for temperatures ranging from 650 to 900 K and in a JSR from 750 to 1050 K. Wang et al. [26] developed a JetSurF2.0 mechanism containing CHX that describes the high-temperature pyrolysis and oxidation of n-alkanes from n-pentane to n-dodecane and the high-temperature chemical reaction of CHX and monoalkylated CHX. This mechanism has 348 species and 2163 reactions. Recently, Zou et al. [27] used SVUV-PIMS and GC+MS to analyze the low-temperature JSR oxidation of CHX at 1.04 bar and *φ* = 0.25, and they developed a detailed oxidation model for CHX. However, the previous research on the CHX model mainly focused on describing the chemical properties of CHX in detail, and it lacked attention to its simplified model.

Some researchers have begun to pay attention to the development of the simplified mechanism of multi-component gasoline-alternative fuels containing CHX. The five-component gasoline-alternative fuel chemical kinetic model developed by Li et al. [21] is composed of n-heptane, isooctane, toluene, DIB, and CHX to predict the ignition delay time. Although this model has been used many times to discuss the influence of gasoline components on the combustion and emissions of gasoline direct-injection engines [28,29], large-scale has caused difficulty in flame-speed verification and three-dimensional numerical simulation. The six-component diesel/gasoline-alternative fuel chemical kinetic model proposed by Raza et al. [6] shows a good mechanism scale (168 species and 680 reactions) and was used to study the combustion characteristics of RCCI. Alternatives with more components have been developed to match a wider range of fuel characteristics [22]. Considering the constraints of computing resources and time cost, these models are difficult for complex three-dimensional engine computational fluid dynamics (CFD) simulations. Furthermore, to the best of our knowledge, few data on laminar burning velocities have been reported on any gasoline surrogate with CHX as one of the mixture components.

As mentioned above, it has been identified that the main challenge for new gasoline engines to achieve high efficiency and low emissions is in-cylinder combustion research. Constructing a simplified chemical kinetics mechanism can reduce the computational cost and space complexity of CFD three-dimensional numerical simulation. In this study, the cyclohexane was chosen as a representative of gasoline naphthenes due to the simple cyclic structure. The CHX mechanism [26] is simplified under a wide range of conditions, and added to the DIB mechanism proposed by Zheng [30] and the TRF mechanism developed by our research group [10]. A five-element simplified mechanism for gasoline suitable for lean combustion is obtained. The mechanism calculations were performed in a zero-dimensional single-zone model and a one-dimensional premixed flame model. The proposed mechanism is evaluated with the ignition delay time, laminar flame velocity, and species distribution data measured in the literature.

## 2. Construction of Chemical Kinetic Model

In this study, a chemical kinetic model of a five-component gasoline fuel substitution mixture containing n-heptane, isooctane, toluene, DIB, and CHX is constructed. The model mainly includes three parts: (a) the TRF sub-mechanism is derived from our previous study of the TRF mechanism [10], and the toluene part of the macromolecular reaction is modified according to the simplified mechanism of Liu et al. [11]; (b) the DIB sub-mechanism is derived from the model constructed by Zheng et al. [30]; and (c) the CHX sub-mechanism is simplified from the JetSurF2.0 mechanism [26]. At present, no simplified version of the CHX mechanism has been found in literature. Therefore, the construction of the CHX sub-mechanism is focused on in this study.

### 2.1. CHX Sub-Mechanism

The JetSurF2.0 mechanism developed by Wang et al. [26] considers the oxidation of various hydrocarbons and is selected as the basic chemical kinetic model of CHX fuel. It contains 348 species and 2163 elementary reactions. The mechanism of JetSurF2.0 is complicated, and its direct use in CFD numerical simulation will promote the increase in computational cost and the decrease in efficiency. Although the available computing power is growing rapidly, the simplification of the detailed mechanism of CHX is essential for the construction of a simplified model of the five-component gasoline-alternative fuel.

In this study, direct relationship graph error propagating (DRGEP) is used to simplify the detailed mechanism into the skeleton mechanism, and then, the rate-of-production analysis (ROP) and the sensitivity analysis (SA) are used to simplify the skeleton mechanism. Details on DRGEP, ROP, and SA are presented in Section 4.2.

#### 2.1.1. Simplified DRGEP

The calculation of the DRGEP method uses the closed homogeneous model. The equivalent ratio is 0.5–1.0, the temperature range is 660–1500 K, and the pressure range is 1–4 MPa. By selecting the absolute error and the relative error of the target parameter in the simplification process, iterative calculations are performed to achieve the simplification goal, and finally, the simplification mechanism that meets the error requirements and has the smallest scale is obtained. The target parameters of the simplified process are selected as the mole fraction of CHX (cC_6_H_12_) and the soot precursor C_6_H_6_, and the ignition delay time. Normalized errors are used as the basis for simplified judgment. A normalized error is defined as

(1) $$\alpha =\frac{\left|D\;-\;S\right|}{\left(R\times \left|D\right|+A\right)},$$

where *D* represents the calculated value of the target parameter for the detailed mechanism, *S* represents the calculated value of the target parameter for the simplified mechanism, *R* represents the relative tolerance, and *A* represents the absolute tolerance. If the normalized maximum error is less than 1.0, then all tolerance settings of the target parameter are met.

The error of the target parameter setting in this simplified process is shown in Table 1.

**Figure 1:**
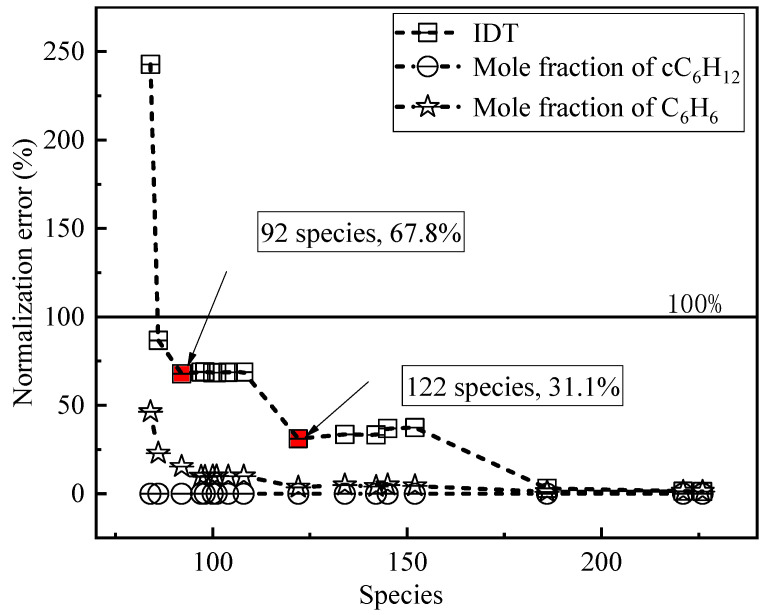
Skeletal mechanism species obtained by direct relationship graph error propagating (DRGEP) and normalized error of the corresponding prediction.

Figure 1 shows the normalized error analysis of the skeleton mechanism species obtained by simplifying the DRGEP method and the corresponding predicted ignition delay time. The DRGEP method generates a series of skeletal mechanisms with different species numbers through the calculation of the original database. For the ignition delay time, the normalization error gradually increases as the number of species decreases. For species composition between 122 and 250, the calculation error is within 50%, and the largest error in the species composition between 50 and 100 can be close to 250%. The normalized error of the mole fraction of cC_6_H_12_ and C_6_H_6_ is within 50%. Notably, the simplification can be considered successful as long as the normalization error of the calculation is guaranteed to be within 100%. However, according to Figure 1, the species is reduced from 122 to 92, and the normalized error of the ignition delay time has more than doubled. Therefore, the simplified mechanism of DRGEP122 is selected as the mechanism of subsequent reaction rate analysis and temperature-sensitivity analysis (SA).

**Figure 2:**
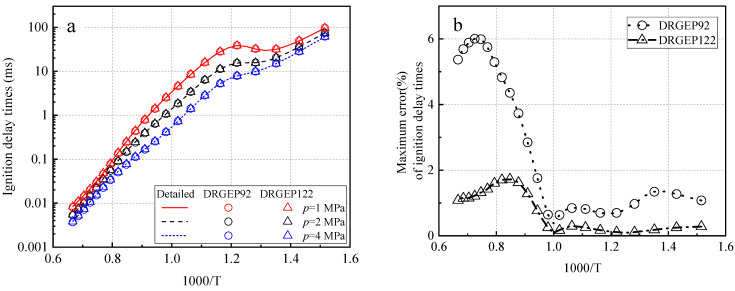
Detailed mechanism and comparison with two skeletal mechanisms (DRGEP92 and DRGEP122) to predict the ignition delay of CHX /air mixture. (**a**) Ignition delay times and (**b**) maximum error of ignition delay times.

The DRGEP analysis method is used to simplify the detailed mechanism of JetSurF2.0, and the two skeleton mechanisms DRGEP92 and DRGEP122 are obtained. DRGEP92 contains 92 species and 550 reactions, and DRGEP122 contains 122 species and 725 reactions. Both are skeletal mechanisms and need to be further simplified. Figure 2 further compares the ignition delay time prediction values of the two framework mechanisms with species 92 and 122, respectively. At an equivalent ratio of 0.5, a pressure of 1–4 MPa, and a temperature range of 660–1500 K, the ignition delay time calculation results of the two mechanisms are in good agreement with the detailed mechanism. The maximum error of the ignition delay is the maximum value of the error of the ignition delay time calculated by the skeleton mechanism and the detailed mechanism at three different pressures in the temperature range of 660–1550 K (shown in Figure 2b). The maximum error of DRGEP92 is obviously higher than that of DRGEP122. This result is understandable because the accuracy of mechanism prediction will be reduced when the component is decreased. Considering the accuracy of calculation, the simplified mechanism of DRGEP122 is selected as the mechanism of subsequent generation rate analysis and temperature SA.

#### 2.1.2. Analysis of the Oxidation Path of CHX

The DRGEP analysis method greatly reduces the simplification pressure of the detailed mechanism, but it is insufficient. This research aims to construct a simplified mechanism. The skeleton mechanism of CHX DRGEP122 has been obtained, including 122 species and 725 elementary reactions. The oxidation path of CHX is analyzed by ROP. The reaction rate of CHX is calculated using the framework mechanism of DRGEP122 under lean conditions (equivalent ratio is 0.5). The initial conditions are as follows: pressure *p* = 2 MPa, temperature is 750 K at low temperature and 1350 K at high temperature, and the threshold of reaction path analysis is 3%.

**Figure 3:**
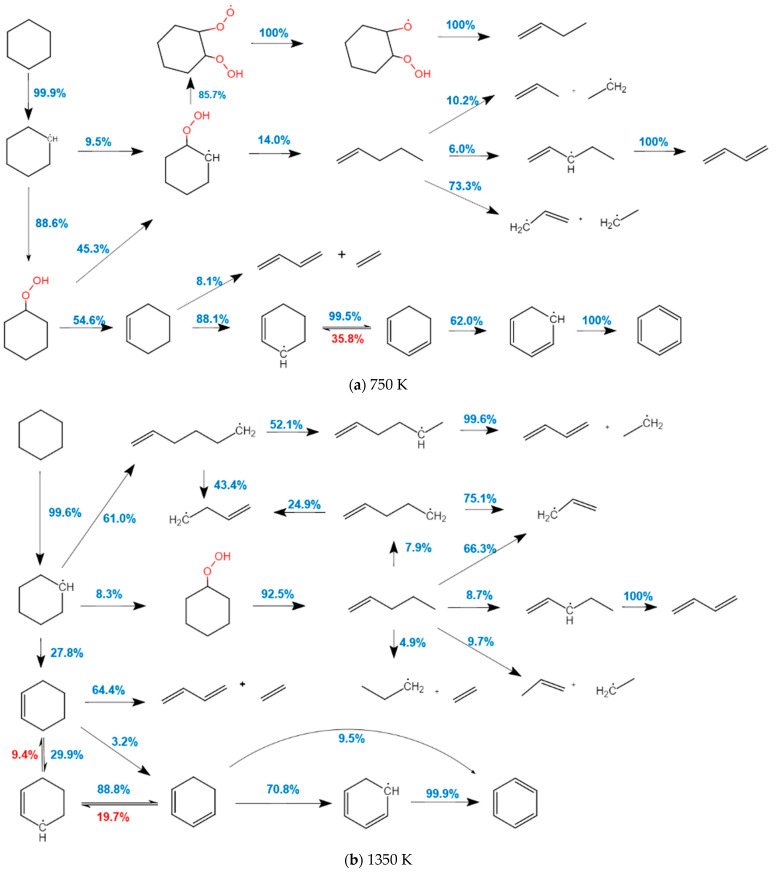
Main oxidation path of CHX. (**a**) 750 K and (**b**) 1350 K.

Figure 3 shows the main path of CHX oxidation. The pressure is *p* = 2 MPa, and the temperature is 750 K for low temperature and 1350 K for high temperature. The blue and red numbers indicate the forward and reverse contributions of each reaction. As shown in Figure 3, the oxidation process of CHX is mainly divided into three parts: (a) Dehydrogenation reactions. At two temperatures, CHX is dehydrogenated and converted to cyclohexyl (cC_6_H_11_). Cyclohexyl and its products undergo continuous dehydrogenation reactions and finally generate benzene rings, which is an important way for CHX combustion to generate PAH; (b) Oxidation reactions. When cyclohexyl radicals are exposed to O_2_, they produce cC_6_H_11_O_2_ (88.6%) and cC_6_H_10_O_2_H-2 (9.5%) at 750 K. (c) Immediate ring-opening reactions. At 1350 K, cyclohexyl radicals open to form 1-hexene radicals (61%) and then undergo a series of reaction cracks to form small molecules such as 1,3-butadiene and ethylene.

**Figure 4:**
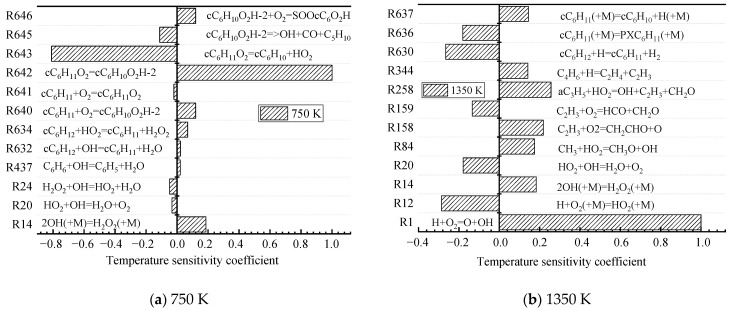
Temperature SA of different temperatures (φ = 0.5, *p* = 2 MPa). (**a**) 750 K and (**b**) 1350 K.

To avoid deleting the reactions that have a low reaction rate and are more sensitive to temperature in the process of generation rate analysis, temperature SA is used to obtain the key reactions at different initial temperatures. Temperature SA can measure the sensitivity of a certain elementary reactions to temperature through the normalized sensitivity coefficient. The negative value indicates the promotive effects of the corresponding reaction on ignition, while the positive one denotes the inhibitive effects. Figure 4 shows that the elementary reactions with larger temperature-sensitivity coefficients are different in different temperature regions. In the low-temperature zone (750 K), the temperature-sensitive reaction is mainly the macromolecular reaction of CHX oxidation. The isomerization of cC_6_H_11_O_2_ to cC_6_H_10_O_2_H-2 (R642) and the cleavage of the C–O bond of cC_6_H_11_O_2_ generate cyclohexene (cC_6_H_10_) and HO_2_ radicals (R643). In the high-temperature zone (1350 K), most of the elementary reactions that are more sensitive to temperature are C_0_–C_4_ small molecule reactions. O atoms and OH radicals (R1) generated by H atoms colliding with O_2_ molecules have a large positive sensitivity coefficient.

**Figure 5:**
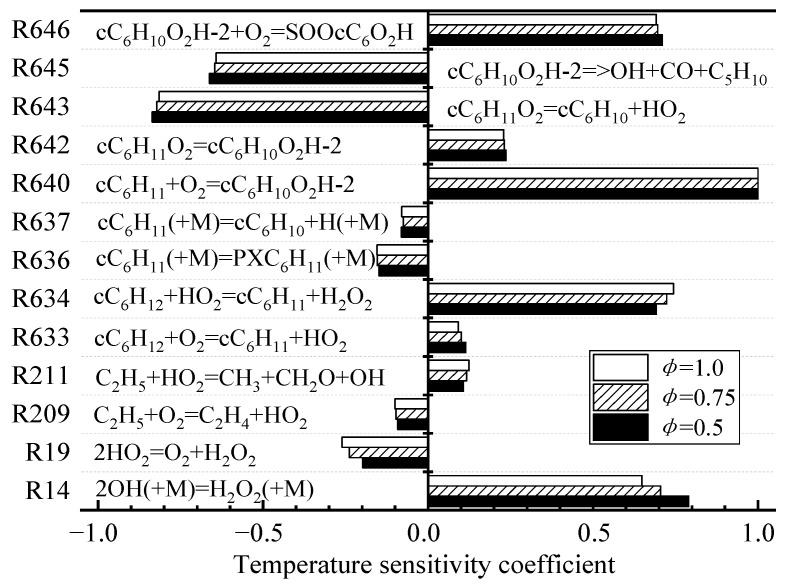
Temperature SA of different equivalence ratios (T = 1000 K, *p* = 2 MPa).

The temperature sensitivity of elementary reactions under different equivalence ratios is analyzed, and some elementary reactions with larger sensitivity coefficients are obtained, as shown in Figure 5. Under different equivalence ratios, large molecule reactions (R634, R640, R643, R645, and R646) and small molecule reactions (R14 and R19) became equally sensitive at temperatures of 1000 K. Notably, the elementary reactions R634 and R640 are less sensitive at 750 and 1350 K (Figure 4), but they have greater sensitivity at 1000 K. The HO_2_ radical in R634 extracts the H atom in CHX, the generated H_2_O_2_ is an important substance for the formation of OH radicals (R19), and R640 is an important path for the oxygenation reaction of CHX.

In this part of the work, the generation rate analysis retains the elementary reactions with a larger reaction rate in the framework mechanism. It also removes the elementary reactions with a smaller reaction rate. So far, a simplified mechanism for CHX has been obtained, including 81 species and 280 elementary reactions (Supplementary Materials Text S1).

### 2.2. Mechanism Merger and Modification

The TRF, DIB, and CHX sub-mechanisms are coupled. The principle of mechanism coupling is to retain all the macromolecular reactions above C_5_ in the five components, delete the repeated elementary reactions between C_1_ and C_4_, and retain the C_0_ molecular reactions in the TRF sub-mechanism. Finally, a simplified chemical kinetic model of CDTRF with 115 species and 414 elementary reactions is constructed (Supplementary Materials Text S2).

After the mechanism is coupled, the prediction accuracy of the mechanism is an issue worthy of attention. Cross-reactions between molecules, changes in component reaction paths and rates, and the fusion of repeated elementary reactions will all lead to changes in ignition delay time and laminar flame velocity. SA can be based on the sensitivity of a component or elementary element reaction to measure the degree of influence of the component or elementary element reaction on the calculation results of the target parameters (ignition delay time and laminar flame speed).

**Figure 6:**
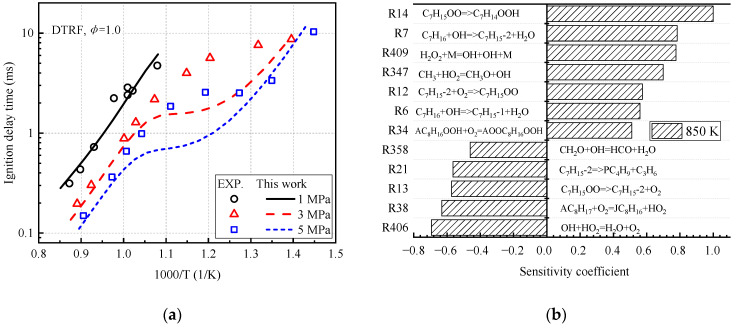
Prediction of ignition delay time and temperature-sensitivity coefficient of DTRF fuel. (**a**) Prediction of ignition delay time and (**b**) temperature-sensitivity coefficient.

The newly constructed simplified mechanism is used to conduct preliminary predictions on the ignition delay time of the four-component gasoline-fuel substitute DTRF (Figure 6). In Figure 6a, Fikri et al. [15] measured the ignition delay time of the four-component alternative fuel value DTRF (25% of isooctane, 20% of n-heptane, 45% of toluene, 10% of DIB, by volume fraction) in the high-pressure shock tube. The experimental conditions were as follows: the pressures are 1, 3, and 5 MPa, and the equivalent ratio was 1. The ignition delay time was predicted using the mechanism constructed in this study. The ignition delay time is defined as the interval from the initial state to the temperature 400 K higher than the initial temperature. The newly constructed mechanism reproduces the ignition of the fuel at 1 MPa. However, it underestimates the ignition delay time in the low-temperature region (T < 1000 K) at 3 and 5 MPa and makes the negative temperature region tend to the high temperature part. Figure 6b further investigates the temperature-sensitive response at the temperature (T = 850 K) at which the maximum error of ignition delay occurs, the pressure is 3 MPa, and the equivalence ratio is 1. The figure shows that the macromolecular reactions (R7, R12, R13, R14) of n-heptane in the system have a greater effect on the ignition delay time. The elementary reaction R14 shows the largest sensitivity coefficient, and C_7_H_14_OOH is generated from C_7_H_15_OO isomerization.

**Figure 7:**
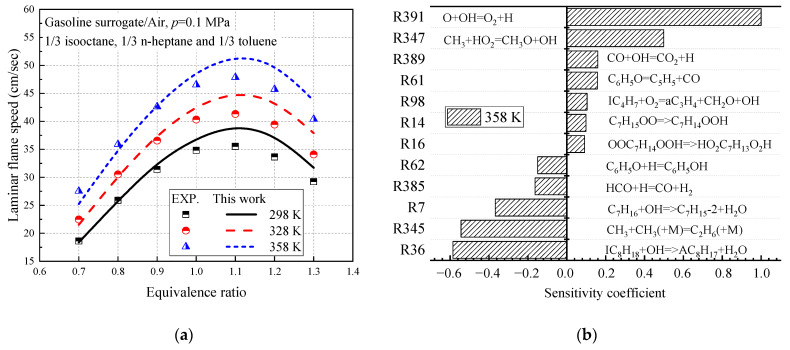
Prediction of laminar flame speed and temperature-sensitivity coefficient of TRF fuel. (**a**) Prediction of laminar flame speed and (**b**) temperature-sensitivity coefficient.

The laminar flame speed of the three-component gasoline-fuel substitute TRF is preliminarily verified in Figure 7. The experimental data for laminar flame speed comes from Sileghem et al. [31]. The laminar flame speed is overestimated in the area of 1.0–1.3 equivalence ratio (Figure 7a). In Figure 7b, at a temperature of 358 K, a pressure of 0.1 MPa, and an equivalence ratio of 1.1, the sensitivity of OH radicals is analyzed. The elementary reaction R391 is found to have a greater sensitivity, which is the main way of generating OH radicals.

According to the predicted results, temperature SA is used to find the key reaction of the newly constructed mechanism under different combustion conditions. Elementary reactions with higher sensitivity (R14. C_7_H_15_OO=>C_7_H_14_OOH and R391. O + OH = O_2_ + H) are selected, and the chemical kinetic parameters of the reaction are modified. The kinetic parameters of R14 refer to the research results of Li et al. [21], while R391 is derived from the mechanism of JetSurF2.0 [26], as shown in Table 2.

After the mechanism is revised, the accuracy of prediction is greatly improved. The revised simplified mechanism ignition delay times and laminar flame speeds prediction will be discussed in the next section.

## 3. Results and Discussion

Whether the constructed chemical kinetic model can be used for the numerical simulation of the combustion process depends on the model’s prediction of the fuel combustion characteristics. The proposed mechanism has verified the combustion characteristics of pure component fuels under lean conditions based on extensive experimental data [16,26,30,31,32,33,34,35,36,37,38,39,40,41,42] (Supplementary Materials Figures S1–S10). This study will verify the combustion characteristics of multi-component alternative fuels from three perspectives—ignition delay time, laminar combustion speed, and concentration distribution of important combustion substances. Table 3 shows the composition ratio of gasoline-alternative fuel components used in this section. The research octane number (RON) of the six alternative fuels ranges from 74 to 95, which can represent most commercial gasoline in the market.

### 3.1. Ignition Delay Times

**Figure 8:**
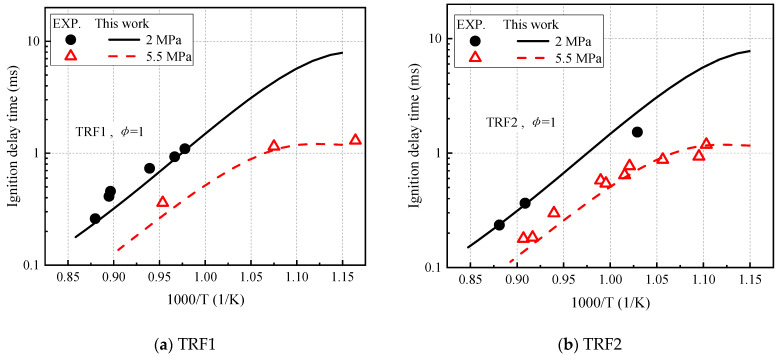
Verification of the ignition delay time of TRF1 and TRF2. (**a**) TRF1 and (**b**) TRF2.

This work chooses the equal-volume closed homogeneous zero-dimensional model to simulate ignition delay. Figure 8 shows the comparison of the ignition delay time calculated by the experiment and the model at different pressures of TRF1 and TRF2. The experimental data was obtained by Gauthier et al. [43] in a low-temperature and high-pressure shock tube. The two experimental pressures were 1.5–2.5 MPa and 4.5–6 MPa. TRF1 data scaled to 2 and 5.5 MPa are denoted as *p*^−0.83^ and TRF2 as *p*^−0.96^. The proposed simplified mechanism is used to calculate the ignition delay time. The simulation results using this mechanism are consistent with the experimental data.

**Figure 9:**
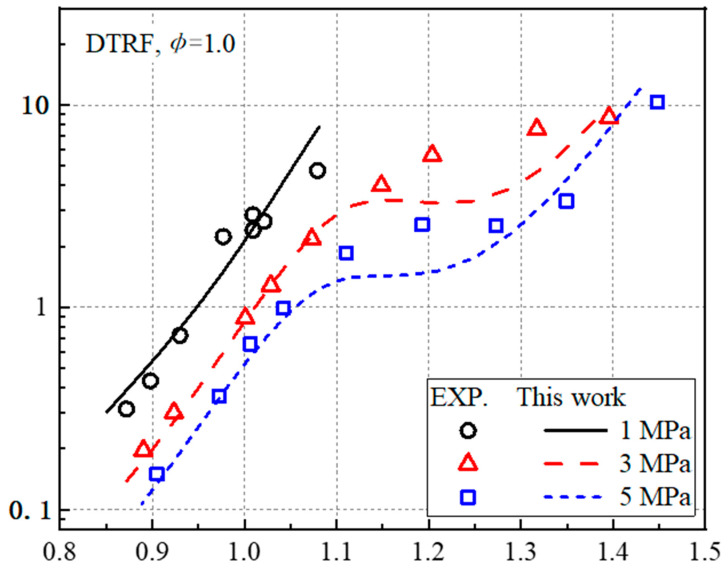
Verification of the ignition delay time of DTRF.

For four-component fuel, Figure 9 shows the comparison between the simulated value of the fuel mixture DTRF described in Table 3 and the experimental value of the high-pressure shock tube by Fikri et al. [15]. The model prediction results in this study are consistent with the experimental results. However, the calculated ignition delay time underestimates the experimental value in the range of 740–910 K. The possible reasons for the pre-ignition in the low temperature region are that the coupling of different sub-mechanisms changes the reaction path and reaction rate of the macromolecular fuel and that there is cross-reaction between the fuel macromolecules.

**Figure 10:**
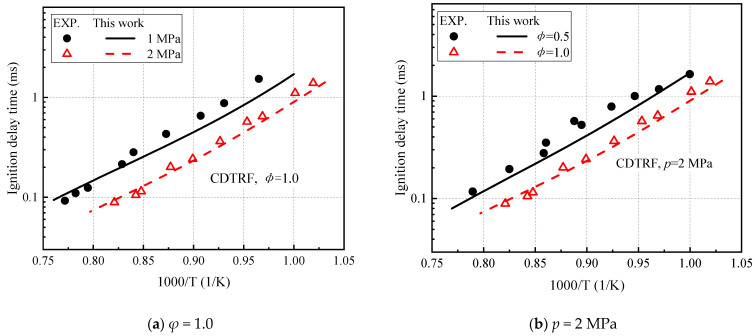
Verification of the ignition delay time of CDTRF. (**a**) *φ* = 1.0; (**b**) *p* = 2 MPa.

Li et al. [21] also measured the high-temperature ignition delay time of CDTRF fuel in a shock tube, as shown in Figure 10. A negative correlation between pressure and equivalence ratio and ignition delay is obtained using our model to predict the ignition delay time of CDTRF fuel. This model reproduces the ignition of the five-component fuel CDTRF over the entire temperature range.

### 3.2. Laminar Flame Speeds

Few experimental studies have been conducted on the laminar flame velocity of gasoline-fuel substitutes with four components. Thus, this study only predicts the laminar flame speed of three-component mixtures.

**Figure 11:**
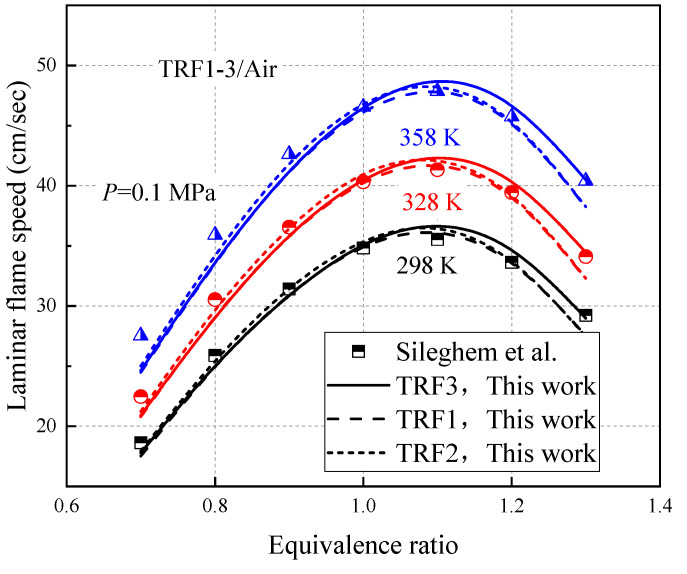
Verification of the laminar flame speed of TRF.

Sileghem et al. [31] found that a mixture of 1/3 isooctane, 1/3 n-heptane, and 1/3 toluene (TRF3) can have a similar laminar flame to the anaerobic gasoline mixture (Exxon 708629-60) speed. Therefore, on the basis of this ratio, the laminar flame speed of toluene reference fuel is measured using the heat flux method on a flat flame adiabatic burner. In this study, three different ratios of toluene reference fuels are used for simulation, and the results are shown in Figure 11. The prediction results of TRF1-3 are consistent with the experimental data of Sileghem. The mechanism constructed in this study can accurately reproduce the flame velocity characteristics of TRF fuel.

### 3.3. Vital Species Distributions in Premixed Flames

**Figure 12:**
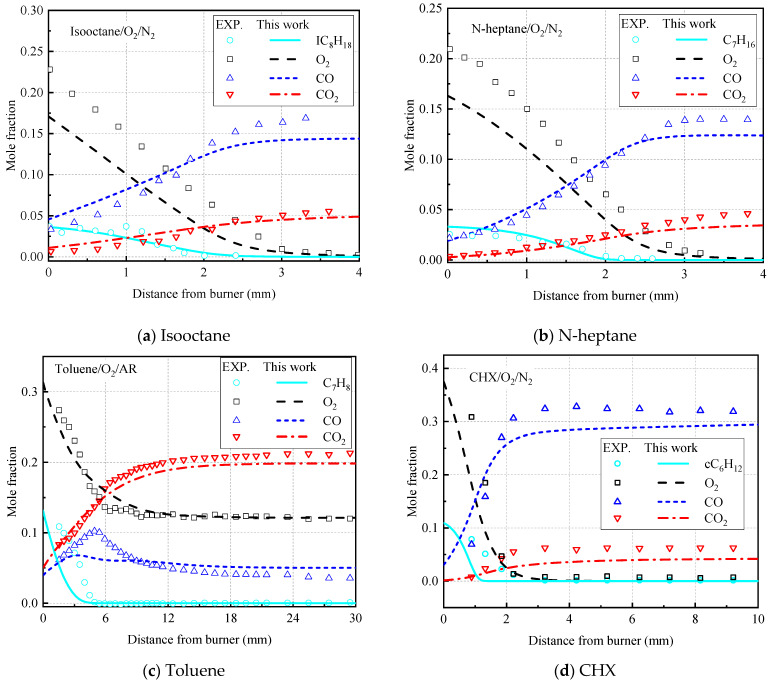
Comparison of the calculation curve of the species in the premixed flame calculated by the experiment [44,45,46] and the model in this study. (**a**) Isooctane, (**b**) N-heptane, (**c**) toluene, and (**d**) CHX.

In addition to the ignition delay time and laminar flame speed, the transport process of fuel molecules should also be described by verifying the distribution of important substances in the premixed flame. In the previous construction process of this mechanism, the distribution of important species in the premixed fuel/air flame was not verified. Therefore, in this study, the important species of premixed flames of isooctane, n-heptane, toluene, and CHX are verified in Figure 12.

El-Bakali et al. [44] used gas chromatography and GC–MS analysis to measure the distribution of the main substances mole fraction of the two fuels in the n-heptane/O_2_/N_2_ and isooctane/O_2_/N_2_ laminar premixed flames. The process is simulated using the mechanism constructed in this study. The initial temperature is 450 K, the initial pressure is 0.1 MPa, the equivalent ratio is 1.9, and N_2_ is used for dilution. The premixed gas inlet velocity is 4.12 cm/s (isooctane), 4.98 cm/s (n-heptane). As shown in Figure 12a,b, the model accurately predicts the mole fraction curve of each substance (IC_8_H_18_, C_7_H_16_, CO, and CO_2_). However, it predicts a lower O_2_ value. We performed a temperature-sensitivity analysis of this system and found that the rapid consumption of the oxidant was mainly attributed to the elementary reaction R391.H + O_2_ = O + OH, showing a high positive sensitivity. The dehydrogenation reaction of isooctane and n-heptane at low temperatures and low pressure produces a large number of H radicals, and the collision of H radicals with O_2_ molecules consumes the oxidant.

The concentration of important substances in the premixed flame of toluene was measured by Li et al. [45] using synchrotron vacuum ultraviolet photoionization mass spectrometry. The initial temperature was 410 K, the initial pressure was 4 kPa, the equivalence ratio was 0.75, Ar was used for dilution, and the premixed gas inlet velocity was 35 cm/s. Figure 12c shows the comparison between the experimental and model calculations of the molar fraction distribution of the main substances in the flame. As observed, the mole fractions of toluene and O_2_ are continuously decreasing, and toluene is completely consumed under lean fuel conditions, while O_2_ is incompletely consumed. The mole fraction of CO calculated by the model does not show an obvious trend of first increasing and then decreasing, while CO_2_ maintains a consistent trend with the experimental data.

Figure 12d shows the comparison between the experiment of Ciajolo et al. [46] and the calculation curve of the species in the CHX premixed flame calculated by the model in this study. The decrease rate of CHX is faster than the experimental value. In general, our model reproduces the distribution of mole fractions of these main substances in a satisfactory manner.

### 3.4. Validation of Real Gasoline

The construction of a chemical kinetic model of gasoline-surrogate fuels aims to reproduce the ignition and oxidation of real gasoline. Therefore, the verification of real gasoline is essential.

**Figure 13:**
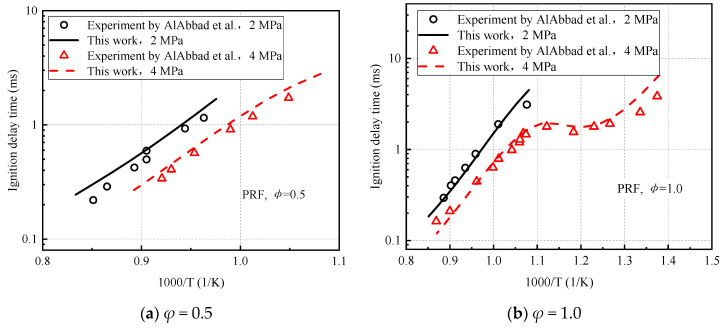
Comparison of ignition delay time calculated by GCI gasoline mixture experiment and model. (**a**) *φ* = 1.0 and (**b**) *φ* = 1.0.

Gasoline compression ignition (GCI) fuel is a customized mixture of Saudi Aramco hydrotreated light naphtha (50% vol.), heavy naphtha (25% vol.), and a high-octane reformate stream (25% vol.). AlAbbad et al. [9] measured the ignition delay time of the low-octane gasoline mixture GCI in a high-pressure shock tube. Figure 13 shows the comparison between the predicted and experimental values of the gasoline-substitute fuel PRF using the same octane number. As expected, the model in this study predicts the ignition delay data of GCI fuel well and shows the negative temperature coefficient region (NTC) consistent with the experiment.

**Figure 14:**
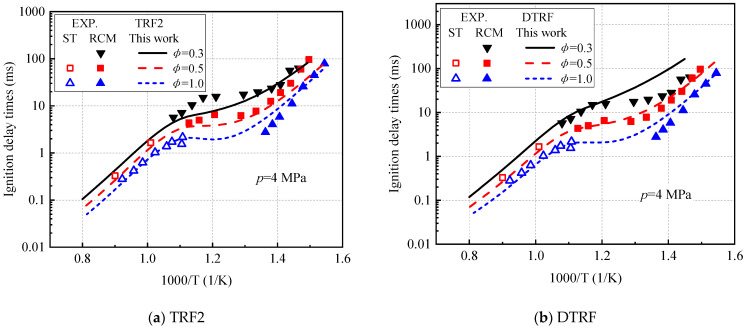
Comparison of ignition delay calculated by RD387 gasoline experiment [43,47] and model. (**a**) TRF2 and (**b**) DTRF.

Gauthier et al. [43] and Kukkadapu et al. [47] measured the high- and low-temperature ignition delay time of mixed gasoline RD387 (RON = 87) in the shock tube and fast compressor, respectively. In this study, the TRF2 fuel with the same octane number is selected for prediction. Figure 14a shows the prediction results under a pressure of 4 MPa, a temperature range of 670–1250 K, lean conditions, and a stoichiometric ratio. In the entire temperature and equivalent ratio range, the TRF2 fuel calculated by this model can predict shock tube and fast compressor data well. The prediction is made under the same conditions using DTRF fuel (Figure 14b). Unfortunately, DTRF fuel below 833 K overestimates the actual gasoline ignition when the equivalence ratio is 0.3, and the performance in the NTC region is not obvious—mainly because the octane number of the DTRF fuel (RON = 94.6) is inconsistent with RD387 gasoline. In addition, the reduction of alkane content in DTRF fuel will inhibit the occurrence of NTC phenomenon [48].

**Figure 15:**
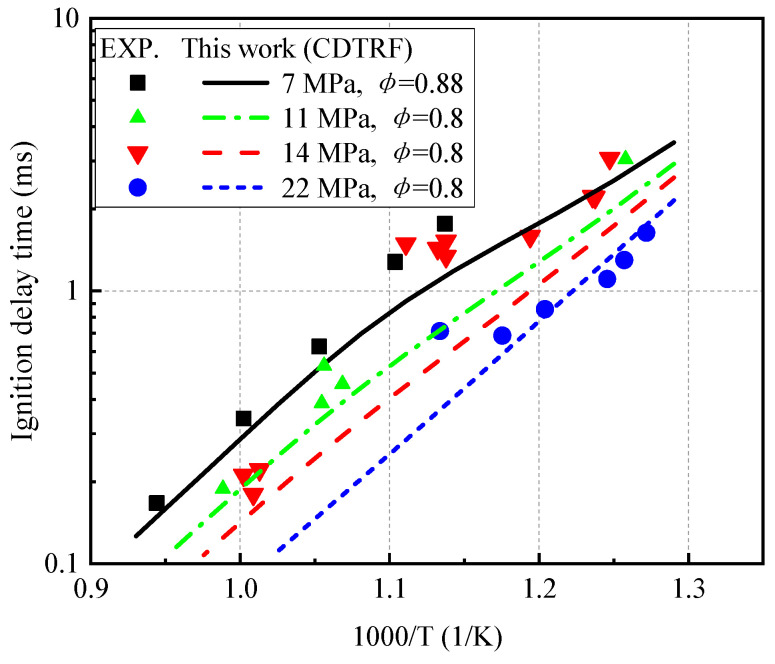
Comparison of ignition delay time between gasoline mixture experiment and model calculation under high pressure.

Davidson et al. [49] used a shock tube to measure the ignition delay time of high-octane gasoline (RON = 105) under high pressure. As shown in Figure 15, this study used gasoline-substitute fuel with an octane number of 95 to simulate the experimental process. Except that the predicted result under 14 MPa pressure underestimates the experimental value, the proposed mechanism reproduces the ignition delay of gasoline under lean conditions.

**Figure 16:**
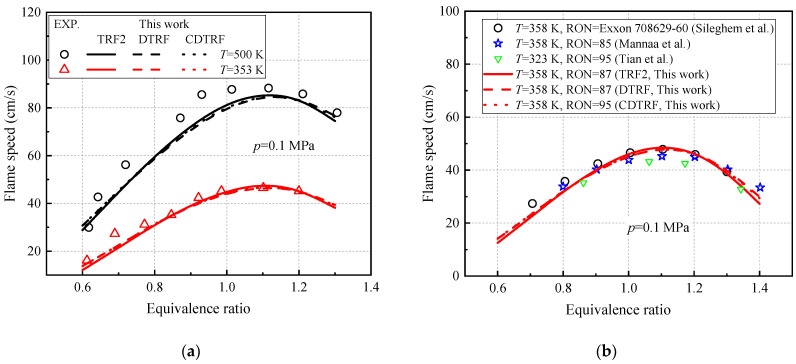
Comparison of laminar flame speed experiment [12,31,50,51] and model calculation of real gasoline. (**a**) Experiment date measured by Zhao et al. [50] at two temperatures of 353 and 500 K. and (**b**) Experiment date measured by Sileghem et al. [31], Mannaa et al. [12], and Tian et al. [51].

For the laminar flame speed of real gasoline, Figure 16a shows the laminar flame speed of real gasoline (CR-87) with an octane value of 87 measured by Zhao et al. [50] at two temperatures of 353 and 500 K. Figure 16b also shows the experimental results of many scholars on gasoline laminar flame speed [12,31,51]. In this study, three fuel mixtures (TRF2, DTRF3, and CDTRF) of the newly constructed model are used to predict the laminar flame speed of gasoline, and the calculated pressure is 0.1 MPa. As shown in Figure 16, the calculated value of the model maintains a consistent trend with the experimental data at 353 and 358 K, and it is slightly lower than the experimental value at 500 K. In general, this model can accurately predict the laminar flame speed of actual gasoline.

## 4. Materials and Methods

### 4.1. Source of Kinetic Mechanisms

In our previous research [10], a chemical mechanism was proposed based on three-component fuel consisting of n-heptane, isooctane, and toluene. Sub-mechanisms were extracted from the TRF mechanism of Andrea et al. [52] and Machrafi et al. [53] using ROP and SA to simplify. The ignition characteristics of this mechanism has been verified in previous studies. In the current work, more combustion characteristics are considered, including ignition delay time, laminar flame speed, and species distribution. The follow-up improvement of the TRF sub-mechanism is studied: (a) Modification of the decomposition of toluene and its reaction with free radicals. The model of Liu et al. [11] simplifies the decomposition, H substitution, and oxidation reaction consumption of toluene fuel molecules. Therefore, the toluene macromolecular consumption reaction has been modified. (b) Supplement kinetic transmission data. In our previous research, TRF laminar flame velocity was ignored, which led to the lack of part of the TRF transmission data. Fortunately, it is supplemented on the basis of the research of Andrae et al. [13] and Park et al. [14].

The simplified mechanism proposed by Zheng et al. [30] is selected as the DIB sub-mechanism. This machine has 50 species and 361 elementary reactions, and it has good predictive performance in terms of ignition and flame speed. Notably, the C_0_–C_3_ mechanism is the core mechanism based on the simplification of the USC-II model, which helps couple the current DIB mechanism with other parts of the fuel sub-mechanism.

The CHX sub-mechanism is obtained by simplifying the JetSurF2.0 mechanism of Wang et al. [26].

### 4.2. Mechanism Simplification Method

#### 4.2.1. Direct Relational Graph Considering Error Propagation

The direct relation graph (DRG) is currently the most common method used in mechanism simplification, and was proposed by Lu and Law [54]. It does not need to involve the numerical calculation of the Jacobian matrix, the calculation amount is small, and the rapid chemical reaction mechanism can be simplified. However, in the DRG simplification process, each species and its strongly coupled species set have the same chance of being selected and retained in the mechanism. The DRG error propagating (DRGEP) was proposed by the researcher, which postulates that the influence of an error introduced by the change in the concentration of a species, or by discarding the species entirely, is damped as it propagates along the graph to reach the targets [55]. In the DRGEP method, the coupling coefficient between two directly related species A and B is estimated as

(2) $$r_{AB}=\frac{\left|\sum_{{i=1,n}_{R}}\nu_{i,A}\omega_{i}\delta_{B}^{i}\right|}{max\left(P_{A}{,C}_{A}\right)}$$

where

(3) $$P_{A}=\underset{{i=1,n}_{R}}{\sum }max\left({0,\nu }_{i,A}\omega_{i}\right)$$

(4) $$C_{A}=\underset{{i=1,n}_{R}}{\sum }max\left({0,-\nu }_{i,A}\omega_{i}\right)$$

where $\nu_{i,A}$ is the net stoichiometric coefficient of species *A* in reaction *i*, and $n_{R}$ is the total number of reversible reactions in the mechanism; and $\omega_{i}$ is the net reaction rate of the *i*-th reaction. If the *i*-th reaction involves species *B*, then $\delta_{B}^{i}$ = 1; otherwise, $\delta_{B}^{i}$ = 0. $r_{AB}$ indicates the strength of the association between component *B* and *A*.

#### 4.2.2. Rate-of-Production Analysis and Sensitivity Analysis

The rate-of-production (ROP) analysis calculates the flux of the consumption reaction and the generation reaction of the components separately, and it also considers the indirect correlation between the components due to the two-step continuous reactions. It can obtain a better simplified mechanism under the same degree of simplification. Sensitivity analysis (SA) can understand the sensitivity of temperature, pressure, and other parameters to a certain reaction, and it can provide a theoretical basis for the simplification of the mechanism. ROP and SA have been introduced in our previous work [56].

*The simplification methods of the three mechanisms all have their own advantages. DRGEP simplification is based on the correlation between species and can greatly reduce the scale of complex mechanisms. Nevertheless, DRGEP can only simplify the detailed mechanism to the framework level. ROP can get a better simplified mechanism at the same degree of simplification, but it is easy to discard those elementary reactions that have a smaller reaction rate but have a greater impact on the system in the process of oxidation-path analysis. SA makes up for the shortcomings of ROP, and uses sensitivity coefficients to reflect the degree of influence of elementary reactions on system parameters and retrieve some of the forgotten elementary reactions.*

### 4.3. Model Calculation Method

In this study, the zero-dimensional single-zone model and the one-dimensional premixed flame model are used for fuel combustion simulation. These functions are included in the simulation software package (Sandia National Laboratories, Albuquerque, NM, USA, 1980). Readers can find relevant introductions in our previous research [56]. We choose the equal-volume closed homogeneous zero-dimensional model (in software) to simulate ignition delay and the one-dimensional premixed flame model (in software) to simulate laminar flame speed and combustion substance concentration.

## 5. Conclusions

(1) In this research, a reduced chemical kinetic model of five-component gasoline substitutes is developed to meet the prediction of gasoline ignition and combustion characteristics under new combustion methods. Based on the construction and validation of the model, the conclusions were obtained as follow: Under lean combustion conditions, the JetSurF 2.0 mechanism is simplified using DRGEP, ROP, and SA, and a CHX simplification mechanism consisting of 81 species and 280 elementary reactions is obtained, which can be accurate to describe the ignition and flame propagation characteristics of CHX.
(2) The model is coupled and modified to form a five-component simplified mechanism of CDTRF consisting of 115 species and 414 elementary reactions.
(3) Under a wide range of conditions, the mechanism’s ignition delay time, laminar flame speed, and the experimental and calculated results of sub-mechanism fuel, multi-component gasoline-surrogate fuel, and real gasoline are compared. The mechanism in this study can accurately reproduce the combustion and oxidation of each component of the gasoline-surrogate fuel mixture and real gasoline.

The results of the study have provided a simplified chemical kinetic model for in-cylinder ignition and combustion in an engine. This model contributes to the 3-D numerical simulation of new engines, thereby reducing the energy and environmental problems caused by traditional fuel combustion. In future studies, the effects of the cyclohexane component of CDTRF on ignition, combustion, and emissions of gasoline-alternative fuels will be investigated.

## Figures and Tables

**Table 1:** Error setting of target parameters.

Target Parameters	Absolute Tolerance	Relative Tolerance
Mole fraction of cC_6_H_12_	1 × 10^−4^	20
Mole fraction of C_6_H_6_	1 × 10^−4^	20
IDT	1 × 10^−6^	10

**Table 2:** Modified key reactions and kinetic parameters.

Reactions	Modification	Kinetic Parameters			Ref.
		A	b	E (J/mol)	
R14. C_7_H_15_OO=>C_7_H_14_OOH	Before	2 × 10^11^	0.0	17,010.0	[10]
	After	1.51 × 10^11^	0.0	19,000.0	[21]
R391. O + OH = O_2_ + H	Before	2 × 10^14^	−0.4	0.0	[10]
H + O_2_ = O + OH	After	2.64 × 10^16^	−0.67	17,041.0	[26]

**Table 3:** Composition of different gasoline-surrogate fuels (by volume fraction).

Fuels	Isooctane (%)	N-Heptane (%)	Toluene (%)	DIB (%)	CHX (%)	RON	Ref.
PRF	77	23	0	0	0	77	[9]
TRF1	63	17	20	0	0	88	[14]
TRF2	69	17	14	0	0	87	[32]
TRF3	33.33	33.33	33.33	0	0	74	[31]
DTRF	25	20	45	10	0	94.6	[15]
CDTRF	30.812	11	38.2	10.342	9.646	95	[21]

## Data Availability

Data are contained within the article.

## Supplementary Materials

The following supporting information can be downloaded. Text S1: CHX simplified mechanism; Text S2: CDTRF simplified mechanism; Figures S1–S5: The five-component model predicts the ignition delay time of isooctane, n-heptane, toluene, DIB, and CHX; Figures S6–S10: The five-component model predicts the laminar flame speed of isooctane, n-heptane, toluene, DIB, and CHX.

## Sample Availability

Samples are not available from the authors.
