# Supplementary material for: Construction of a Chemical Kinetic Model of Five-Component Gasoline Surrogates under Lean Conditions
Source: Molecules. 2022 Feb 6;27(3):1080. doi: 10.3390/molecules27031080 (PMC8840295; doi:10.3390/molecules27031080)
Supplement: Supplementary file 1 [file molecules-27-01080-s001.zip › Figures S1¿CS10.pdf]

molecules

MDPI

Figures S1–S5. The five-component model predicts the ignition delay time of isooctane, n-heptane, toluene, DIB and CHX

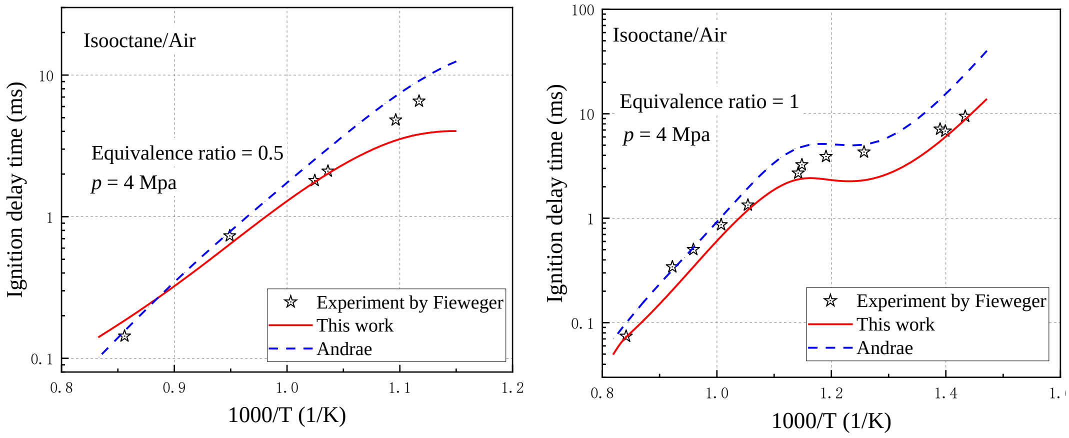

a. φ=0.5

b. φ=1.0

Figure S1. Predicted ignition delay times against experimental measurements in shock tube tests for isooctane at various equivalences. Experiment data is from Ref.[1], the model constructed in this article and Andrae[2].

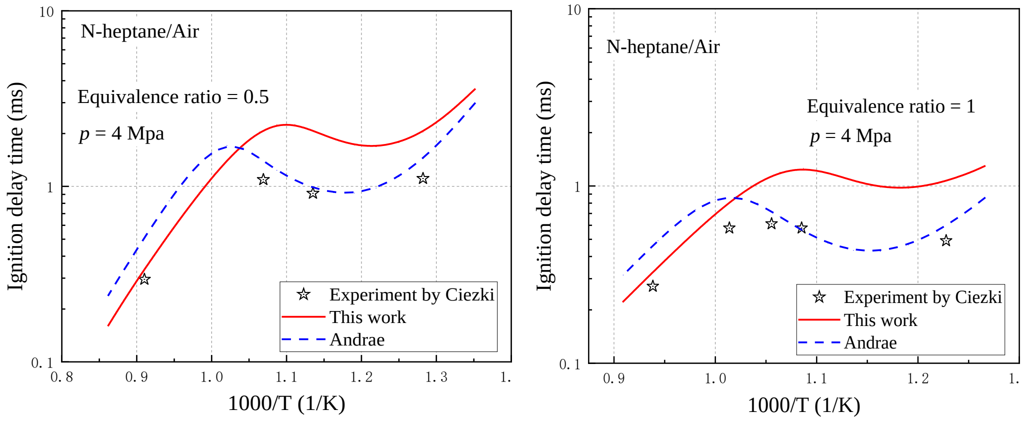

a. φ=0.5

b. φ=1.0

Figure S2. Predicted ignition delay times against experimental measurements in shock tube tests for n-heptane at various equivalences. Experiment data is from Ref.[3], the model constructed in this article and Andrae[2].

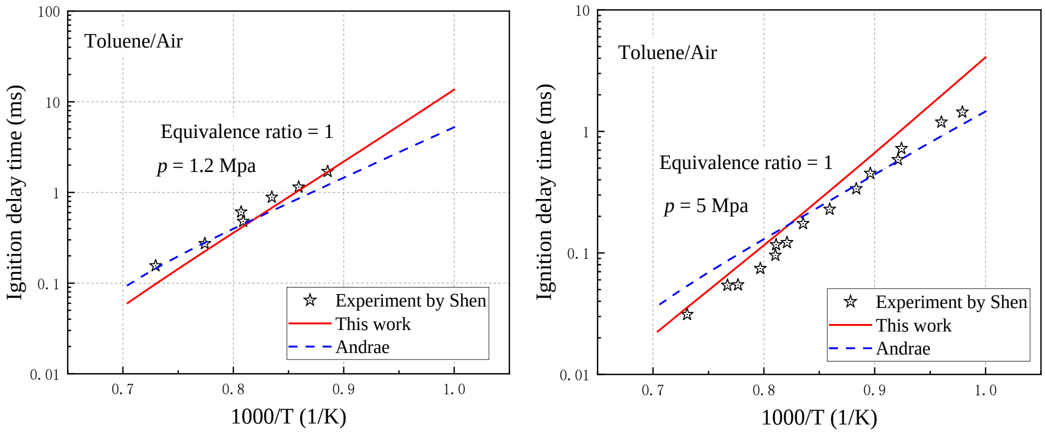

a. *p*=1.2 Mpa b. *p*=5.0 Mpa

Figure S3. Predicted ignition delay times against experimental measurements in shock tube tests for toluene at various presures. Experiment data is from Ref.[4], the model constructed in this article and Andrae[2].

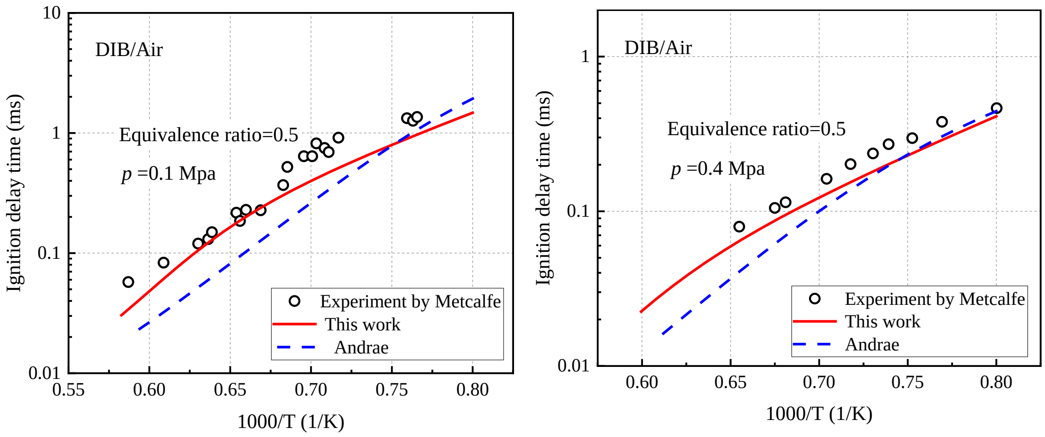

a. *p*=0.1 Mpa b. *p*=0.4 Mpa

Figure S4. Predicted ignition delay times against experimental measurements in shock tube tests for DIB at various presures. Experiment data is from Ref.[5], the model constructed in this article and Andrae[2].

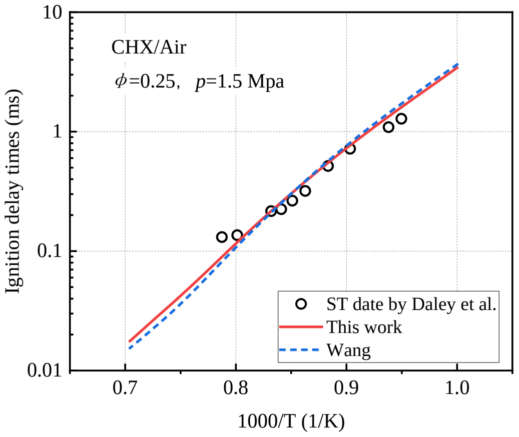

a. φ=0.25， p=1.5 Mpa

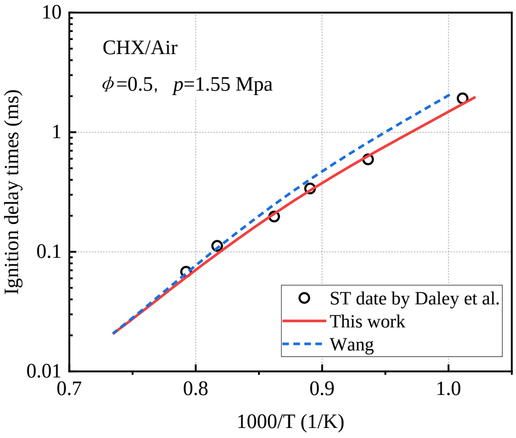

b. φ=0.5， p=1.5 Mpa

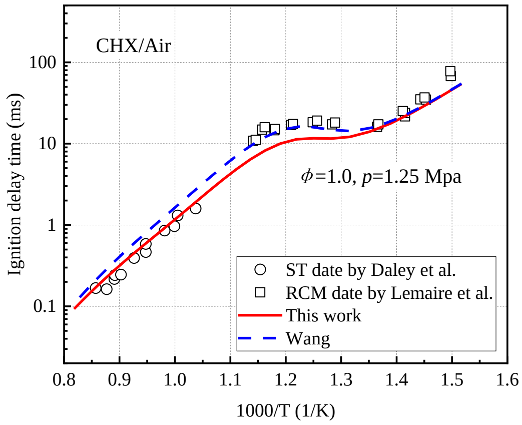

c. φ=1.0， p=1.25 Mpa

Figure S5. Predicted ignition delay times against experimental measurements in shock tube and RCM tests for CHX at various equivalences. Experiment data is from Ref.[6,7], the model constructed in this article and Wang et al.[8].

Figures S6–S10. The five-component model predicts the laminar flame speed of isooctane, n-heptane, toluene, DIB and CHX

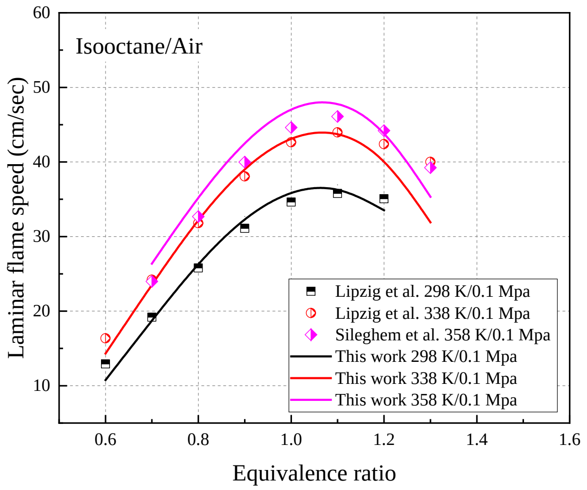

Figure S6. Comparison of experimental and computed laminar burning velocities for isooctane-air mixture. Experiment data is from Ref.[9,10].

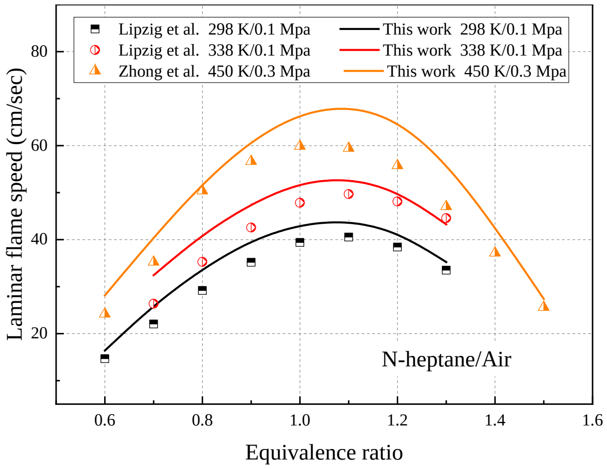

Figure S7. Comparison of experimental and computed laminar burning velocities for n-heptane-air mixture. Experiment data is from Ref.[9,11].

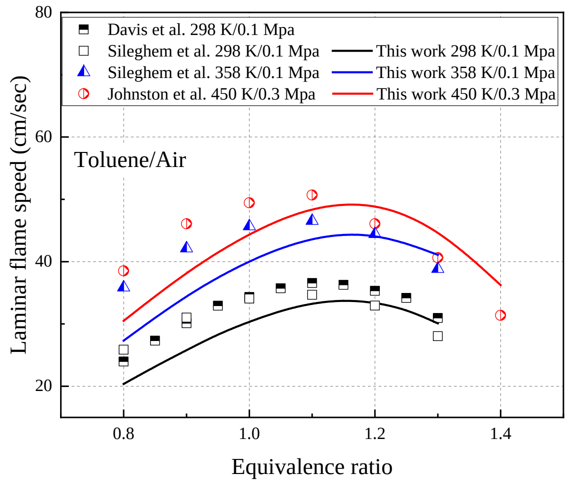

Figure S8. Comparison of experimental and computed laminar burning velocities for toluene-air mixture. Experiment data is from Ref.[9,12,13].

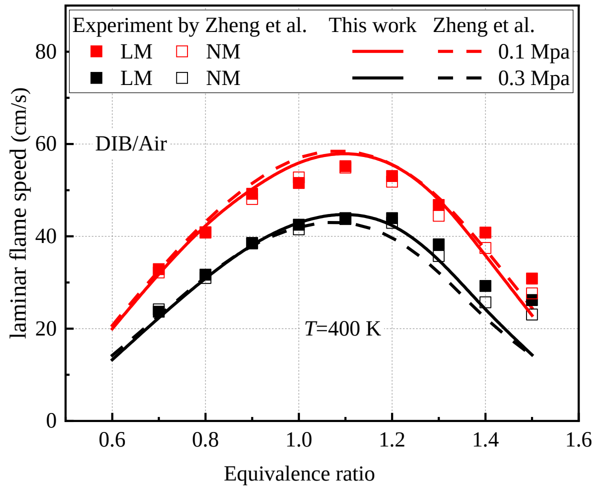

Figure S9. Comparison of experimental and computed laminar burning velocities for DIB-air mixture. Experiment data is from Ref.[14], the model constructed in this article and Zheng et al.[14].

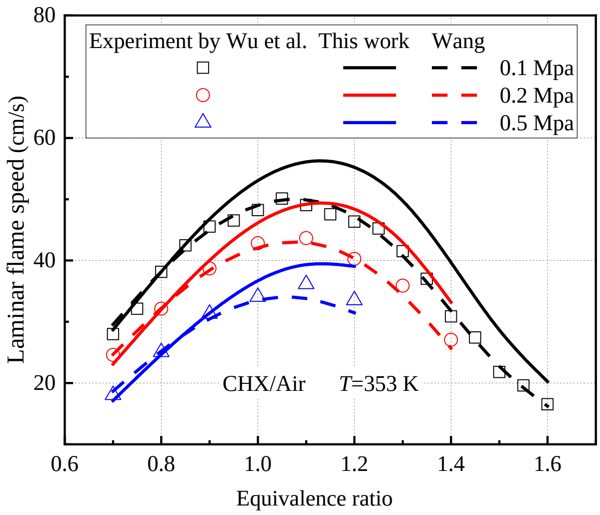

Figure S10. Comparison of experimental and computed laminar burning velocities for CHX-air mixture. Experiment data is from Ref.[15], the model constructed in this article and Wang et al.[8].

References

1. Fieweger, K.; Blumenthal, R.; Adomeit, G. Self-ignition of S.I. engine model fuels: A shock tube investigation at high pressure. *Combustion and Flame* **1997**, *109*, (4), 599–619. https://doi.org/10.1016/S0010-2180(97)00049-7.
2. Andrae, J.C.G. Development of a detailed kinetic model for gasoline surrogate fuels. *Fuel* **2008**, *87*, 2013–2022. https://doi.org/10.1016/j.fuel.2007.09.010.
3. Ciezki, H.; Adomeit, G. Shock-tube investigation of self-ignition of n-heptane-air mixtures under engine relevant conditions. *Combustion and Flame* **1993**, *93*, (4), 421–433. https://doi.org/10.1016/0010-2180(93)90142-P.
4. Shen, H.-P.S.; Vanderover, J.; Oehlschlaeger, M.A. A shock tube study of the auto-ignition of toluene/air mixtures at high pressures. *Proceedings of the Combustion Institute* **2009**, *32*, 165–172. https://doi.org/10.1016/j.proci.2008.05.004.
5. Metcalfe, W.K.; Pitz, W.J.; Curran, H.J.; Simmie, J.M.; Westbrook, C.K. The development of a detailed chemical kinetic mechanism for diisobutylene and comparison to shock tube ignition times. *Proceedings of the Combustion Institute* **2007**, *31*, 377–384. https://doi.org/10.1016/j.proci.2006.07.207.
6. Lemaire, O.; Ribaucour, M.; Carlier, M.; Minetti, R. The production of benzene in the low-temperature oxidation of cyclohexane, cyclohexene, and cyclohexa-1,3-diene. *Combustion and Flame* **2001**, *127*, 1971–1980. https://doi.org/10.1016/S0010-2180(01)00301-7.
7. Daley, S.M.; Berkowitz, A.M.; Oehlschlaeger, M.A. A shock tube study of cyclopentane and cyclohexane ignition at elevated pressures. *International Journal of Chemical Kinetics* **2008**, *40*, 624–634. https://doi.org/10.1002/kin.20353.
8. Wang, H.; Dames, E.; Sirjean, B.; Sheen, D.A.; Tango, R.; Violi, A.; Lai, J.Y.W.; Egolfopoulos, F.N.; Davidson, D.F.; Hanson, R.K.; Bowman, C.T.; Law, C.K., Tsang, W.; Cernansky, N.P.; Miller, D.L.; Lindstedt, R.P. A high-temperature chemical kinetic model of n-alkane (up to n-dodecane), cyclohexane, and methyl-, ethyl-, n-propyl and n-butyl-cyclohexane oxidation at high temperatures, JetSurF version 2.0, September 19, 2010 (http://web.stanford.edu/group/haiwanglab/JetSurF/JetSurF2.0/index.html).
9. van Lipzig, J.P.J.; Nilsson, E.J.K.; de Goey, L.P.H.; Konnov, A.A. Laminar burning velocities of n-heptane, iso-octane, ethanol and their binary and tertiary mixtures. *Fuel* **2011**, *90*, 2773–2781. https://doi.org/10.1016/j.fuel.2011.04.029.
10. Sileghem, L.; Alekseev, V.A.; Vancoillie, J.; Van Geem, K.M.; Nilsson, E.J.K.; Verhelst, S.; Konnov, A.A. Laminar burning velocity of gasoline and the gasoline surrogate components iso-octane, n-heptane and toluene. *Fuel* **2013**, *112*, 355–365. https://doi.org/10.1016/j.fuel.2013.05.049.
11. Zhong, B.-J.; Peng, H.-S.; Zheng, D. The effect of different class of hydrocarbons on laminar flame speeds of three C7 fuels. *Fuel* **2018**, *225*, 225–229. https://doi.org/10.1016/j.fuel.2018.03.126.
12. Davis, S.G.; Wang, H.; Breinsky, K.; Law, C.K. Laminar flame speeds and oxidation kinetics of benene-air and toluene-air flames. *Symposium on Combustion* **1996**, 26, (1), 1025–1033. https://doi.org/10.1016/S0082-0784(96)80316-X.
13. Johnston, R.J.; Farrell, J.T. Laminar burning velocities and Markstein lengths of aromatics at elevated temperature and pressure. *Proceedings of the Combustion Institute* **2005**, *30*, 217–224. https://doi.org/10.1016/j.proci.2004.08.075.

14. Zheng, D.; Zhong, B.-J.; Xiong, P.-F. Experimental study on laminar flame speeds and chemical kinetic model of 2,4,4-trimethyl-1-pentene. *Fuel* **2018**, *229*, 95–104. https://doi.org/10.1016/j.fuel.2018.05.011.
15. Wu, F.; Kelley, A.P.; Law, C.K. Laminar flame speeds of cyclohexane and mono-alkylated cyclohexanes at elevated pressures. *Combustion and Flame* **2012**, *159*, 1417–1425. https://doi.org/10.1016/j.combustflame.2011.11.012.
